# Functional morphology and biomechanics of arthropods

**DOI:** 10.1007/s00359-023-01621-1

**Published:** 2023-02-22

**Authors:** Chao Wan, Stanislav Gorb

**Affiliations:** 1 Department of Mechanics, School of Aerospace Engineering, Beijing Institute of Technology, Beijing, China; 2 Functional Morphology and Biomechanics, Zoological Institute, Kiel University, Kiel, Germany; 3 Tangshan Research Institute, Beijing Institute of Technology, Tangshan, China

**Keywords:** Functional morphology, Biomechanics, Movement, Arthropods

## Abstract

Representatives of arthropods, the largest animal phylum, occupy terrestrial, aquatic, arboreal, and subterranean niches. Their evolutionary success depends on specific morphological and biomechanical adaptations related to their materials and structures. Biologists and engineers have become increasingly interested in exploring these natural solutions to understand relationships between structures, materials, and their functions in living organisms. The aim of this special issue is to present the state-of-the-art research in this interdisciplinary field using modern methodology, such as imaging techniques, mechanical testing, movement capture, and numerical modeling. It contains nine original research reports covering diverse topics, including flight, locomotion, and attachment of the arthropods. The research achievements are essential not only to understand ecological adaptations, and evolutionary and behavioral traits, but also to drive prominent advances for engineering from exploitation of numerous biomimetic ideas.

## Introduction

As the largest phylum of animals, arthropods live all over the world occupying terrestrial, aquatic, arboreal, and subterranean niches. The distinct living environments resulted in evolving numerous complex adaptations associated with movement and other mechanical functions. Schroeder et al. (2018) proposed that such functions as locomotion, mechanical detection, adhesion, sound production, communication, etc., result from specific combinations of cuticle-derived exoskeleton. Although the exoskeleton in arthropods is mainly formed by three main constituents (chitin, proteins, and lipids), hierarchically structured materials with tremendous diversity of properties have evolved resulting in different morphological features and specific biomechanical characteristics. It has been previously reported by Vincent and Wegst (2004) that insect cuticles have seven-order range of Young’s modulus, ranging from 1 kPa for soft cuticles, like in the case of intersegmental membranes, to 20 GPa for sclerotized cuticle, such as in the beetle elytra. The distinct elastic properties greatly satisfy the mechanical requirements of the cuticles for realizing a wide range of biomechanical and other physiological functions.

To better understand the extraordinary mechanical behaviors of biological materials, numerous studies have been performed using imaging techniques, experimental measurements, and numerical simulations. Interesting recent findings include the presence of gradient elasticity in adhesive tarsal setae of beetles caused by inhomogeneous resilin distribution (Peisker et al. 2013), anti-friction properties of porous fluid-filled sand hopper cuticle surface (Wan and Gorb 2020), and fracture resistance of multi-layered locust semi-lunar process cuticle (Wan and Hao 2020), to name a few.

In addition to the reinforcement mechanism of the functional biological materials, scientists and engineers are also interested in revealing how specific materials and structures of arthropods are related to their properties and functions. However, investigations should not just report some statistical relations between anatomical features and performance characteristics, to speculate about potential reasons. Instead, multidisciplinary methodology should be implemented to explore the key mechanisms and principles behind their outstanding performance. This is quite a difficult task, because many different features in organisms are simultaneously adapted, to achieve multi-optimized properties, rather than a single one. The reason for multi-optimized properties is that organisms are usually adapted to different environmental stresses and sometimes face unexpected risks. The protective shell of the mussel *Mytilus edulis* (though it is not representative of Arthropoda) is a good long-term studied example to explain this viewpoint. Its shell is a multi-layered biomineralized system consisting of several different materials for protecting its soft body inside. Compared to the outer prismatic calcite in the shell, the inner nacre layer is stronger, although from the engineering point of view should be actually deposited outside the shell for providing more effective protection against external impacts. However, this disagrees with the natural situation that nacre is situated on the inner side of the shell rather than on the outer side. Both acid etching test and mechanical loading tests (including static loading and dynamic impact) were performed in our previous research (Wan et al. 2019), revealing an interesting compromise of the mussel shell in that the current pattern of outside prismatic calcite and inside nacre can provide optimal multi-protection against both mechanical and chemical stresses. Perhaps, in view of just mechanical protection, the current pattern of layers is not the best solution, but the present evolutionary optimized distribution of layers offers the mussel sufficient multi-protective abilities against complex environmental stresses (like acid attack of dog whelk gastropods and mechanical attack of decapod crabs).

Due to the complexity of the research object of biological materials, modern imaging and characterization techniques must be simultaneously used to decode the mysteries of the arthropods. General technologies include imaging techniques (micro-CT, transmission electron microscope, scanning electron microscope), compositional identification (X-ray energy spectrometry, Fourier transform infrared spectroscopy, Raman spectrometry, immunohistological staining, confocal laser scanning microscopy), mechanical testing (nanoindentation, atomic force microscopy, universal material testing technology, adhesion force testers, and various tribometers), movement capture (high-speed videography, videography under synchrotron radiation light source), and numerical simulations (finite element modeling, computational fluid dynamics simulation, fluid–solid coupling modeling, multibody dynamics simulation).

In this special issue of the *Journal of Comparative Physiology A*, some recent research on functional morphology and biomechanics of arthropods will be presented to link morphology and biomechanics of materials and structures to their mechanical functions, such as flapping flight, legged locomotion, and gripping.

## Wings and flight

The first topic of this special issue is insect flight and associated structures. O’Callaghan and Lehmann (2023) studied the flight movement of the tiny thrips *Gynaikothrips ficorum*, which has a bristled wing rather than a membranous wing. They quantified the aerodynamic performance of upscaled artificial bristled wings during wing flapping, scored its circulation during wing translation, and investigated its fate at the stroke reversals. Their results indicated that the aerodynamic force of the bristled wing is approximately 9% less than the common insect wing of solid membranes. This work clarifies the flow conditions of the bristled insect wing and deepens our understanding of flying in tiny insects.

The cross-veins in the hind wing of locust *Locusta migratoria* were quantitatively investigated by Zhao et al. (2022). They evaluated the cross-veins at different regions of the locust hind wing using uniaxial tensile tester, scanning electron microscope, and finite element modeling method. Four cross-vein types were found at different sites of the hind wing, which exhibit similar tensile stiffness and various bending compliances. The naturally distributed cross-veins with different mechanical properties can be attributed to their biomechanical functions, like lift force production, damage reduction, and resistance against other environmental impacts.

Finally, Shen et al. (2022) examined the morphological and mechanical characteristics of the wings of butterfly *Tirumala limniace* using scanning electron microscopy, tensile experiments, finite element, and computational fluid dynamics simulation. Specific orientation of fiber alignment was found in the multi-layered structures of the wings and the small deformation of the wings under various loads verified the stability of the butterfly wing structure. All the results confirmed the effect of wing veins in maintaining the flight performance of the butterfly.

## Legs and locomotion on solid ground

The second topic of this special issue is ground mobility or terrestrial locomotion of insects. Zong et al. (2022) filmed the take-off, flight, and landing of flea beetles *Altica cirsicola* on a configurable angled platform to investigate their landing behavior. They found three in-flight modes for the flea beetle, which significantly affected take-off speed, acceleration, and the deploying time of wings.

Dürr and Mesanovic (2023) compared three species of stick insects (Phasmatodea) with strong different antennae–leg length ratios and further explored how relative limb length varied between sexes and throughout postembryonic development. They found that relative limb length directly related with the near-range exploration effort, and the antennae and front legs serve as complementary effect. The limb-to-body proportion is species characteristic while both antennae and front leg had different growth rate.

Bruns et al. (2022) built up a novel experimental methodology for analyzing 3D-escape behaviors of insects and quantified the spatial orientation performance of house crickets *Acheta domesticus*. The comparison between their measurements and literature results validates the usefulness of this new experimental setup in studying various strategies of animal spatial orientation.

## Gripping

The third topic of this special issue focuses on the gripping ability of arthropods. Salerno et al. (2022) compared the attachment ability to plants in three Coleoptera species with different claw shapes. The attachment forces on various substrates were measured using traction force experiments or centrifugal force test. It was indicated that the attachment performance on the plant surface with trichomes is stronger and directly related to the trichome stiffness. Special claws-associated structures are revealed in the coccinellid beetles that are able to grasp plants surfaces covered with soft unbranched trichomes.

Furthermore, Winand et al. (2022) investigated the gripping forces of stick insect *Sungaya inexpectata* on multiple substrates with various roughnesses and directions to address the question why the majority of adult insects developed paired claws. They found that the gripping forces decreased even on smooth substrates, when the claws were removed. It revealed the collective effects of different attachment devices during locomotion.

It is well known that the morphology of the benthic stream insects is well adapted for locomotion function under these specific environmental conditions. In the paper by Ditsche et al. (2023), the role of femora in attachment of the mayfly larvae *Ecdyonurus *sp. under stream conditions was investigated using microscopic techniques, 3D printing, and drag-lift measurements of artificial femoral profiles in a wind tunnel. The authors found that the widened femora of *Ecdyonurus sp.* aid in the negative lift force generation. Additionally, lift can be regulated by the animals through adjusting tilt of their femora or changing distance to the ground.

## Outlook

Taken together, the above contributions collected in this special issue aim to reveal the novel morphological and biomechanical characteristics for some biomechanical functions of arthropods. We hope this special issue will not only deepen our understanding of the key principles behind biological functions, but also provide some innovative idea for future bioinspired technologies in engineering and industry.
